# Muscle activation patterns around knee following neuromuscular training in patients with knee osteoarthritis: secondary analysis of a randomized clinical trial

**DOI:** 10.1186/s40945-022-00140-7

**Published:** 2022-07-07

**Authors:** Shahzada Aadil Rashid, Mohammad Ejaz Hussain, Pooja Bhati, Zubia Veqar, Adila Parveen, Insha Amin, Shahzada Mudasir Rashid

**Affiliations:** 1 Center for Physiotherapy & Rehabilitation Center, Jamia Millia Islamia, New Delhi, India; 2 Faculty of allied health sciences, SGT University Gurugram, Gurgaon, India; 3 Faculty of Physiotherapy, SGT University Gurugram, Gurgaon, India; 4 Division of Veterinary Biochemistry, Faculty of Veterinary Sciences (SKUAST-K), Shuhama, Alusteng, Srinagar, Kashmir 190006 India

**Keywords:** Co-contractions, Knee pain, Neuromuscular, Exercises, Genus varum

## Abstract

**Objective:**

To compare the effects of neuromuscular training (NMT) to a quadriceps strength training (QT) program on co-contraction index (CCI) of knee muscles in patients with knee osteoarthritis (OA).

**Methods:**

Sixty-six knee OA patients with varus malalignment were recruited from the physiotherapy outpatient department of the university. After baseline measurements, they were randomly assigned into two groups: NMT (*n* = 33) and QT (*n* = 33). Patients in NMT group received neuromuscular exercises whereas QT group received conventional strengthening exercises for a period of 12 weeks, three times per week. Electromyographic (EMG) activity of quadriceps, hamstring and gastrocnemius muscle was evaluated during treadmill walking before and after 12 weeks of intervention period and CCI of medial quadriceps-medial hamstring (med QH), lateral quadriceps-lateral hamstring (lat QH), medial quadriceps-medial gastrocnemius (med QG) and, lateral quadriceps and lateral gastrocnemius (lat QG) was calculated.

**Results:**

There was a significantly greater reduction in CCI of med QH (*p* = 0.02) and lat QH (*p* = 0.01) in the NMT group than the QT group. Whereas both NMT and QT led to statistically similar reductions in CCI of med QG (*p* = 0.08) and lat QG (*p* = 0.66).

**Conclusion:**

Findings of this study suggest that NMT led to a greater reduction in CCI of knee muscles than QT which indicates that enhanced sensori-motor control attained by NMT could reduce knee loading in knee OA patients with varus malalignment.

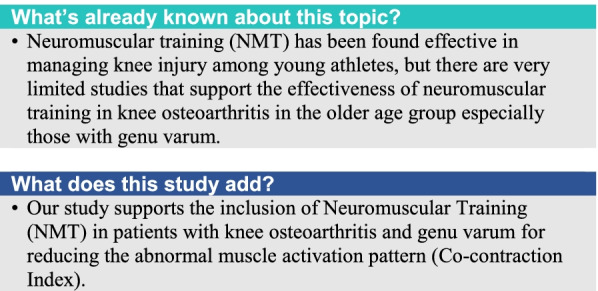



## Introduction

Knee osteoarthritis (OA) is a common chronic joint disorder which leads to pain, loss of function and reduced quality-of-life [1]. Medial joint compartment is more prone to OA than the lateral compartment of knee presumably due to greater loads (60–80% of the entire load) passing through it throughout the gait cycle [2]. Disproportionate loading of the medial compartment is considered to be an important reason for the development of varus malalignment in the knee joint. One of the common findings in knee OA is weakness of quadricep muscles [3], that makes quadriceps strengthening to be an important component of the exercise program designed to manage OA conservatively. However, isolated quadriceps strengthening remains to be ineffective in alleviating pain in patients with varus mal-alignment [4], nor does it help in reducing the knee adduction movement in those with neutral or varus misaligned knees [5]. Existing literature [6, 7] suggest that muscular co-contractions are frequently experienced by knee OA patients during walking and other functional activities. These co-contractions increase compressive loads at the knee joint surface and accelerates structural progression of the disease [7].The co-contraction index (CCI) represents a weighted ratio of the EMG signal intensities obtained from two antagonistic or synergistic muscles with reference to the maximum EMG signal intensity achieved during maximum voluntary muscle contractions (MVCs) [8]. This index is typically raised in knee OA patients and has been found to be associated with increased knee loads and knee joint adduction moment in these patients [9]. Moreover, it has been shown that increased knee joint loads contributes to the development of knee OA [10]. In the management of knee OA, conventional quadriceps strengthening program primarily aims to improve the muscle output, rather than targeting the biomechanics of the medial compartment’s knee load [11]. Consequently, an alternative exercise programs are required focusing on knee joint loading patterns that bring down symptoms in patients with medial compartment OA and varus impairment.

Neuromuscular exercise being relatively a broad category of exercises include functional, proprioceptive, agility, or perturbation training. Neuromuscular exercises are typically performed in functional weight-bearing positions emphasizing quality and efficiency of movement, as well as alignment of the trunk and lower limb joints [12]. Previous research [13] demonstrates that neuromuscular exercise improves functional performance, proprioception and muscle strength. Neuromuscular exercises may enhance activation of certain muscle groups by improving the position of the knee in relation to the hip and ankle which are capable of counteracting the increased knee adduction moment [14]. Such muscle groups include the hip adductors [11], the tensor fascia lata, lateral hamstrings, quadriceps and lateral gastrocnemius [15–18]. Moreover, considering the fact that varus malalignment along with ineffective dynamic muscle stability can lead to lateral thrusting of the knee during early stance phase of walking (which is associated with increased risk of disease progression) [19], enhanced control of lateral knee movement by neuromuscular exercises could be considered beneficial. Neuromuscular exercises are primarily administered for the prevention and rehabilitation of knee injuries in young athletes [20].The training program contains sports specific tasks such as jumping and cutting activities, which are not suitable for sedentary older individuals having knee OA. However, only a few such studies that utilizes task directed NMT for sedentary knee OA patients are available [21–23].

A few studies conducted on the effects of neuromuscular training (NMT) in patients with knee OA [21–23] had shown equivocal results. Addition of NMT to a conventional rehabilitation program led to a significant improvement in outcomes of balance and physical function in knee OA patients in the study of Gandave Pranita and Twinkle [23], whereas NMT was found to be equally effective as conventional exercises for improving pain and physical function in the study of Bennell et al. [21]. To the best of our knowledge, no study has examined the effect of NMT on knee muscle activation patterns. Therefore, considering paucity of literature regarding the effects of neuromuscular exercises on activation patterns of knee musculature, the present study was aimed to investigate and compare the effects of NMT to a conventional quadriceps training (QT) on the co-contraction index (CCI) of knee musculature in knee OA patients with varus malalignment. We hypothesized that NMT will be significantly more effective than QT in decreasing the CCI of knee musculature in patients with knee OA with varus malalignment.

## Material and methods

The present study contains secondary data from a randomized clinical trial [24] and is reported in accordance with CONSORT statement (checklist of information to include when reporting randomized trial).

### Participants

The present study was a single blinded parallel group randomized clinical trial conducted at Centre for Physiotherapy and Rehabilitation Sciences, Jamia Millia Islamia, New Delhi, India between January 2014 and February 2015.Sample size was calculated from a previous study [3] utilizing software G power using differences in root mean square of vastus medialis muscle in response to NMT at an α of 0.05 and power of 95%. Based on these estimates, a sample size of 62 subjects (31 in each group) was found to be necessary. Ethical approval was obtained from the Human Research Ethics Committee of the university vide No: 12–6146. A written informed consent was obtained from each participant for their participation in the study. Study procedures were explained in detail to all the participants prior to commencement of the research procedures. A total of 66 patients were recruited into the study via advertisement through the university website, posters displayed in the university campus/ adjoining areas, and through physician’s referral from the university’s medical Centre. Eligibility criteria for the present study was in accordance with the study of Bennell et al. [21]. Inclusion criteria for the present study were: both male and female subjects aged ≥45 years with medial knee OA [Kellgren-Lawrence (KL) grade ≥ 2] [25] and varus malalignment were included if they had knee pain over the past week (≥ 2.5 on 10 cm visual analogue scale), pain/tenderness over medial joint line of the knee, medial joint space narrowing grade < lateral joint space narrowing grade, medial tibiofemoral osteophyte grade ≥ lateral tibiofemoral osteophyte grade [26]. Exclusion criteria for the present study were: patients using intra-articular or oral corticosteroids within past 6 months or 4 weeks respectively (these drugs have an effect on outcomes of knee OA i.e. pain which may have an influence on muscle activation), post-surgical knee, knee or hip joint replacement surgery, tibial osteotomy (these conditions could alter the biomechanics of the knee joint), any other condition which influences lower limb function, current or past non-pharmacological treatment including physiotherapy or massage or acupuncture (as these treatments could have an effect on muscle activation patterns), participation in any form of exercise therapy within the past 6 months (It could confound the effect interventions utilized in the present study), uncontrolled hypertension (exercise is contraindicated in such conditions), history of cardiovascular disease (to avoid adverse events), pregnancy (to avoid any adverse events), or cognitive impairments (as these patients may not follow directions and instructions), and unable to ambulate without a gait aid (use of gait aid may alter knee biomechanics and muscle activation patterns) were excluded. Those on non-steroidal anti-inflammatory drugs, chondroitin, or glucosamine drugs were allowed to participate in the study (since it would have been an ethical issue if we excluded such patients). Inclusion and exclusion criteria were followed from a previous study on knee OA patients that included NMT as the form of intervention [21].

### Procedures

All eligible participants were randomly allocated into either the NMT group or the QT group by simple random sampling. A lottery method (wherein a researcher randomly picks numbers, with each number corresponding to a subject or item, in order to create the sample) was used to assign participants into two equal groups i.e. NMT (*n* = 33) and QT (*n* = 33). Sixty-six small chits were placed in a box, and participants were allowed to take out the chits. The numbers were written in chits, and the odd number chits were assigned to NMT group and even number chits were assigned to QT group. The investigator who enrolled the participants and generated the random allocation sequence was different from the one who randomly allocated the participants into the groups. Participants were informed about the purpose of the study, testing procedure for data acquisition, duration of the study and the exercise protocol to be followed. Only one knee was considered for evaluation purposes as it reduced inconvenience cost to the participants during the laboratory testing time. In case of bilateral symptoms, the most symptomatic knee was chosen and if the symptoms were found to be equal, right knee was selected for the assessment purpose [12]. Following general demographic assessment, surface electromyography (EMG) of the quadriceps; hamstring and gastrocnemius muscle was recorded during self-paced treadmill walking. This assessment was part of a larger test session which included assessment of pain, physical function and gait parameters which are included in the primary paper [24].

### Electromyography

CCI calculated from EMG data was the secondary end-point for the present study (primary end-points are reported in Rashid et al. [24]) For surface EMG, prior to silver-chloride (AgCl) electrode placement, skin was shaved, abraded and cleaned as per the standard recommendations [27]. Inter-electrode distance was kept to be approximately 2 cm [28]. Electrodes were placed on skin in bipolar arrangement for medial and lateral quadriceps muscles, hamstring muscle and gastrocnemius muscle as per the recommended guidelines (Table 1) [29].

**Table 1:** Placement of electrodes for quadriceps, hamstring and gastrocnemius muscle

Muscles	Placement of electrodes
Biceps femoris (LH)	35% of the distance from the ischial tuberosity to the lateral side of the popliteus cavity, starting from the ischial tuberosity.
Semitendinosus (MH)	36% of the distance from the ischial tuberosity to the medial side of the popliteus cavity, starting from the ischial tuberosity
Vastus lateralis (LQ)	94 mm of the distance (mm) along a line from the superior lateral side of the patella to the anterior superior iliac spine, starting from the patella.
Vastus medialis (MQ)	52 mm of the distance from superior medial side of the patella along a line medially oriented at an angle of 50 degree with respect to the anterior superior iliac spine.
Gastrocnemius medialis (MG)	50% of the distance from the medial side of the popliteus cavity to the medial side of the achilles tendon insertion, starting from the achilles tendon.
Gastrocnemius lateralis (LG)	60% of the distance from the lateral side of the popliteus cavity to the lateral side of the Achilles tendon insertion, starting from the achilles tendon

*LH* lateral hamstring, *MH* medial hamstring, *LQ* lateral quadriceps, *MQ* medial quadriceps, *MG* medial gastrocnemius, *LG* lateral gastrocnemius

Surface EMG was recorded at the sampling rate of 1200 Hz, using a band-pass filter between 350 and 25 Hz [30]. (Lab chart software, AD Instruments, New Zealand). For maximum voluntary isometric contraction (MVIC) testing, participants were seated with the knee in 90^0^ of flexion for assessing the quadriceps muscles. For hamstring muscle, they were positioned in prone lying with knee flexion of 20^0^ and for gastrocnemius, they were asked to stand on a single limb [31]. Three MVIC tests were performed for each muscle with a rest period of 30 s between the tests. Subjects were instructed to hold the extension or flexion contraction for approximately 5 s against the resistance offered by the examiner for quadriceps and hamstring muscle contraction respectively. For gastrocnemius, subjects standing on one leg were asked to raise the heel to maximum and hold for 5 s. Participants were asked to walk at a self-selected pace on a motorized treadmill. An attendant was instructed to stay behind participant to prevent any fall or injury while the participants were asked to walk at self-selected pace on treadmill. Clear instructions were given to the participant to report any alleviation of symptoms and were asked to take rest till the symptoms are relieved. Safety stop cord was clipped onto participant’s body for safety purpose. The EMG was recorded once the patient was comfortably walking on the treadmill. The EMG data recorded from the vastus medialis (VM), vastus lateralis (VL), lateral hamstring (LH), medial hamstring (MH), medial gastrocnemius (MG) and lateral gastrocnemius (LG) from 100 ms prior to heel strike to maximum knee flexion. EMG amplitude (root mean square) of the muscles recorded during the test was normalized by their respective MVICs. Calibrated electrogoniometer was placed on lateral aspect of the testing knee joint with the help of straps to record joint excursion in order to locate the heel strike to maximum knee flexion phase. The high definition camera captured the phases of gait and was placed 2 m perpendicular to the knee joint. Heel strike was synchronized with the motion captured from the video camera and deflection on the electrogoniometer graph. Maximum knee flexion was synchronized with the graph of electrogoniometer showing peak knee flexion. CCI for each muscle group [(medial and lateral quadriceps-hamstring (med and lat QH), medial and lateral quadriceps and gastrocnemius (med and lat QG)] was determined using the equation described by Rudolph et al. [32].

$\frac{EMGS}{EMGL}\times \left( EMGS+ EMGL\right)$, where EMGS was activity level of the less active muscle and EMGL was activity level of the more active muscle. The index was multiplied by the sum of the activity found in the two muscles. Analysis of the EMG data was performed by an investigator blinded to group allocation of participants and details of the interventions.

### Intervention

Interventions are reported in accordance with the TIDieR checklist. Participants were subjected to either QT or NMT for a period of 12 weeks, three sessions per week. Exercise sessions were supervised and each session lasted for around 30–40 min in both the groups. The total session duration in minutes per group for 12 weeks ranged from 1080 to 1440. Both the interventions were performed under the supervision of the same physiotherapist (SAR) at the gymnasium of the university. QT mainly consisted of quadriceps muscle strengthening performed at an intensity of 40 to 60% of 1 repetition maximum (1RM) (progressed from 40% of 1 RM to 50% of 1RM in the initial 6 weeks and from 50 to 60% of 1RM in the last 6 weeks of the intervention period) [21]. 2–3 sets of 8–12 repetitions were performed for each exercise involved in the protocol 1 repetition maximum (1 RM) was determined from repetitions to fatigue and was estimated using the equation proposed by Brzycki [33].

*Predicted*1*RM* = *W*/(1.0278 − 0.0278 ∗ *X*), where W = weight in kg lifted, X = the number of repetitions performed.

Quadriceps strengthening was executed using weight plates on quadriceps table and elastic therabands. Other exercises in QT included quadriceps isometrics and resistance band exercises; strengthening of ankle dorsiflexors, ankle plantar flexors, knee flexors, knee extensors, hip abductors, hip adductors, hip external rotators and internal rotators. Progression was achieved by increasing the number of sets, the duration of the hold phase of the exercise, and the ankle weight or elastic band resistance, as guided by the physiotherapist.

**Fig. 1:**
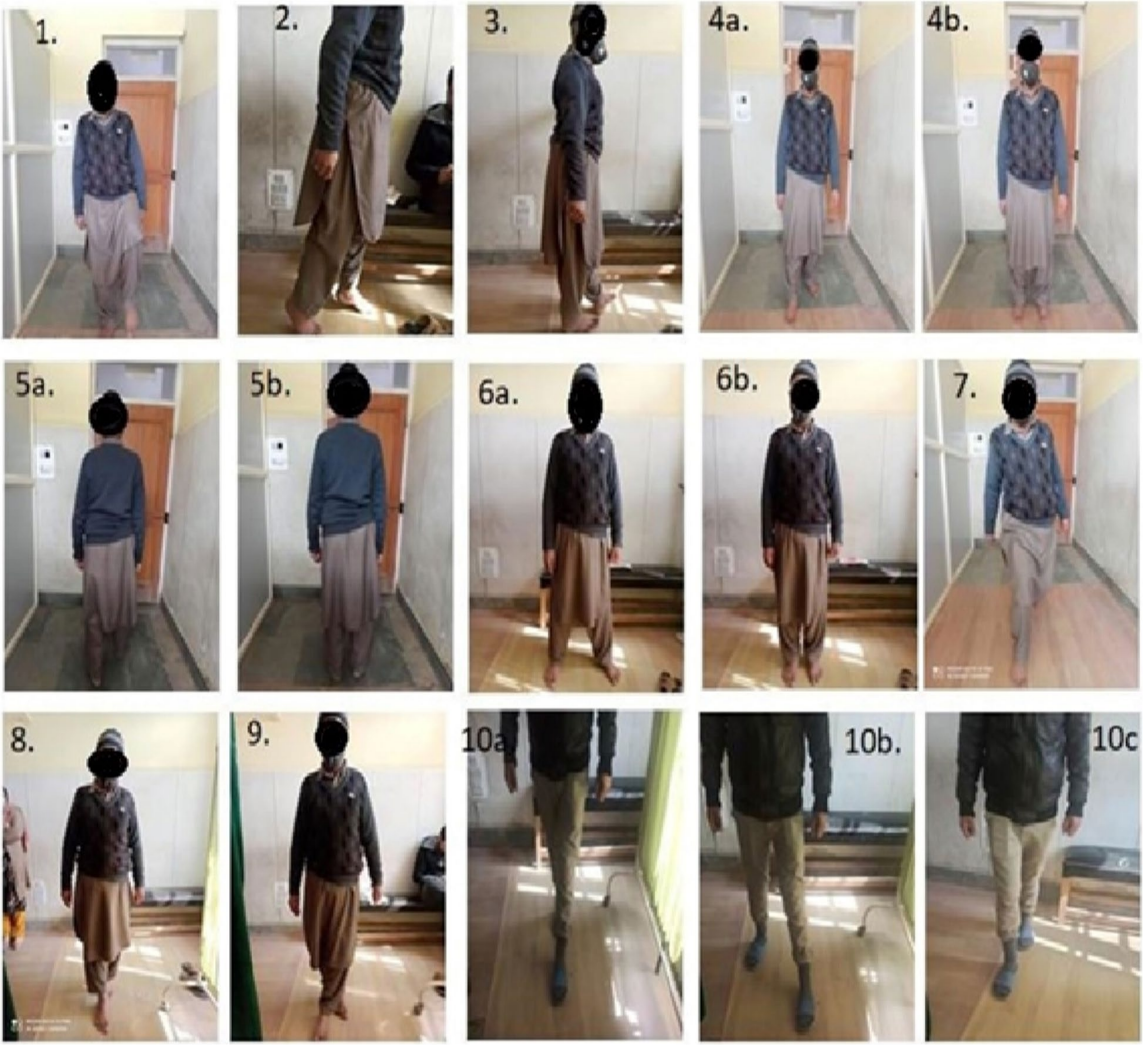
(1-10c) 1.High Knee March 2. Toe Walking 3. Heal Walking 4a.4b. Wedding March. 5a,5b. Backward Wedding March. 6a, 6b. Side Stepping 7. Knee over Toe Position 8. Tandem Walk. 9. Cross Over Walk 10a, 10b, 10c. Modified Grapevine Walk

NMTincluded exercises for agility, balance and proprioception keeping in view the alignment of trunk while performing lower limb exercises [34]. Initially the participants were allowed to do the exercises that were less challenging like wedding march, side stepping, backward wedding march, toe walking, heel walking, followed by semi-tandem walk, tandem walk, modified grapevine, cross-over walk, knee over toe position (Fig. 1; 1-10c). Stability training on firm surface was initiated and when the participants were able to hold single limb support for more than 10 seconds comfortably graded theraband stability trainer foam pads were introduced. Details of the exercises involved in the NMT program are presented in Table 2. The duration of each exercise of NMT program ranged from 3 to 5 minutes or was ended until exhaustion within 5 minutes. Each session was of 30–40 minutes duration. Progression, as determined by the physiotherapist, was provided by varying the repetitions, direction, and velocity of the movements by increasing the load and/or changing the support surface. NMT involved the use of stability trainers and uneven surfaces.

**Table 2:** Exercises involved in the neuromuscular training program

Neuromuscular exercises	
Knee over toe position	In sliding and stepping lunge on even and uneven surface, with or without support
Wedding march	Take a step forward and slightly to one side with the main foot, unite the trailing foot with driving foot; interchange driving foot
Backward wedding march	As above, stepping backward
Side stepping	Walk sideways with the leading foot stepping sideways and trailing foot following to leading foot, then repeat the same in opposite direction
High knees march	March forward while bending hip around 90^0^
Semi-tandem walk	Heel of one foot lands just in front of but, slightly medial to great toe of opposite foot
Tandem walk	Heel of one foot lands just in front of opposite foot and walk in straight line.
Modified grapevine	One foot stepping sideways, the trailing foot lands behind the driving foot, again leading foot stepping sideways and the trailing foot lands in front of the driving foot; repeat the cycle; interchange the driving foot and repeat in opposite direction
Cross-over walk	March forward with each foot crossing midline of the body
Toe walking	Advancing forward on toes
Heel walking	Advancing forward on heels
Stability training	Theraband stability trainer foam pads (green, blue, black or silver)

NOTE: Progression of each exercise was determined by the physiotherapist and was provided by varying the repetitions, direction, and velocity of the movements by increasing the load and/or changing the support surface

### Statistical analysis

**Fig. 2:**
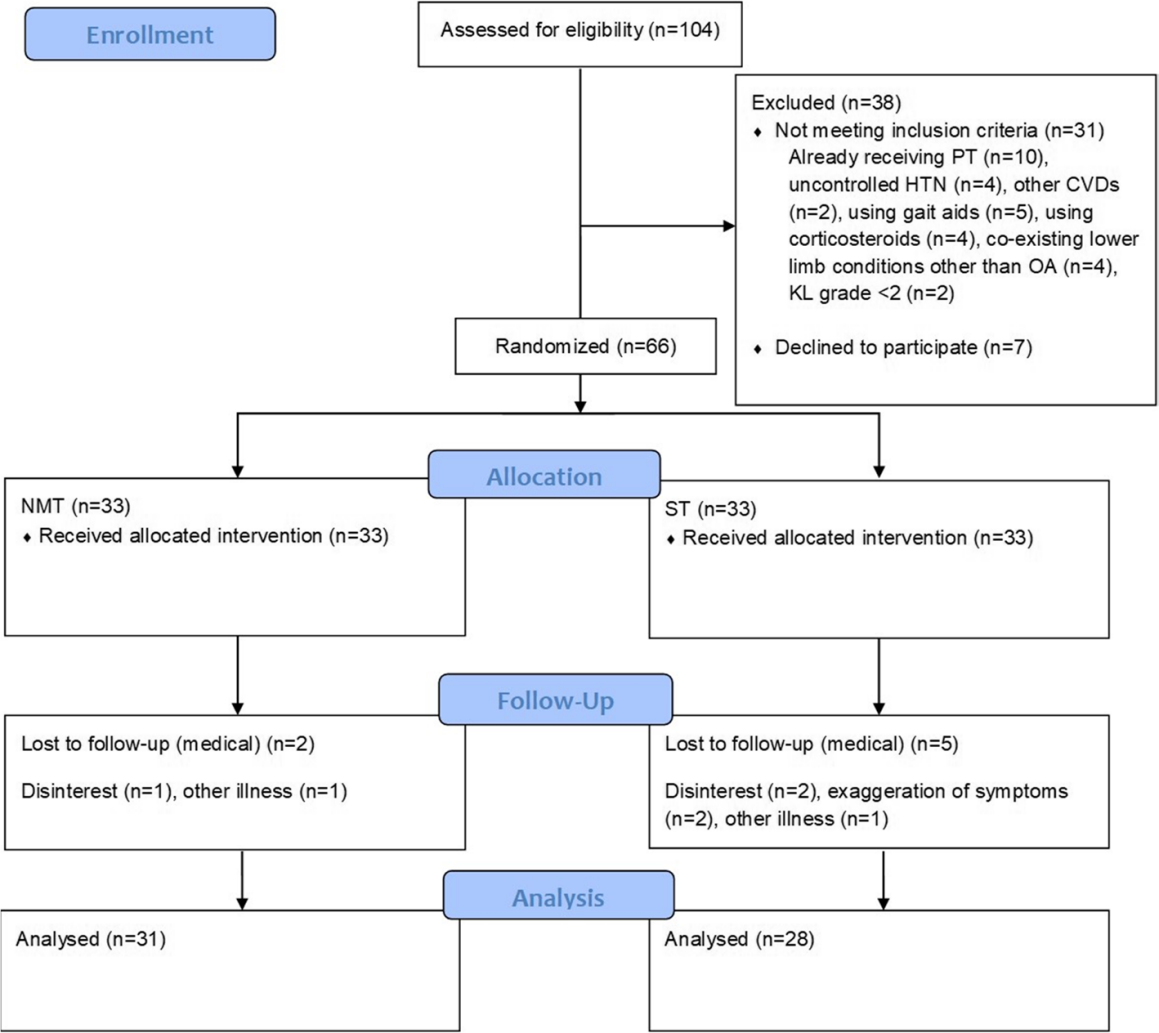
Participant flow through the study. PT: physiotherapy; HTN: hypertension, CVDs: cardiovascular diseases; OA: osteoarthritis; KL grade: Kellgren-lawrence; NMT: neuromuscular training; ST: strengthening program

Data of only those participants which completed the study (*n* = 59) was analyzed (Fig. 2). Data analysis was performed using the software IBM SPSS version 20. The distribution of data was evaluated using Shapiro-Wilk test. Non-normal data underwent log-transformation prior to further analysis. Between group comparison of demographic and outcome variables at baseline was undertaken using an independent *t* test. The CCI after completion of NMT and QT was compared between the groups using an independent sample *t* test. Within group analysis was performed using paired *t* test. *p* values < 0.05 were considered statistically significant for the present study.

## Results

### Baseline characteristics of participants

Initially, sixty-six participants were recruited, of which 7 participants dropped out during the course of the study (Fig. 2). The final analysis was performed on 31 subjects in the NMT and 28 subjects in the QTgroup. Demographic and baseline characteristics of all participants are presented in Table 3. There were no significant differences between the groups at baseline for outcome variables (Table 3).

**Table 3:** Comparison of demographic and outcome variables between the groups at baseline

Variables	NMT (***n*** = 31)	QT (***n*** = 28)	***P***
Age (years)	57 ± 6.8	54.6 ± 9.3	
BMI (kg/m^2^)	27.5 ± 3.8	28.6 ± 2.6	
VAS (0–10 cm)	5.2 ± 1.01	4.9 ± 0.8	
**Gender, n (%)**			
Male	22 (71%)	11 (39.3%)	
Female	9 (29%)	17 (60.7%)	
**KL grades, n (%)**			
2	10 (32.3%)	15 (53.6%)	
3	21 (67.7%)	13 (46.4%)	
4	–	–	
**Muscle activation patterns (CCI)**			
med QH	.58 ± 0.17	.54 ± 0.14	.38
lat QH	.88 ± 0.31	.84 ± 0.28	.29
med QG	.61 ± 0.14	.59 ± 0.20	.14
lat QG	.75 ± 0.27	.71 ± 0.33	.32

*med QH* medial quadriceps-medial hamstring, *lat QH* lateral quadriceps-lateral hamstring, *med QG* medial quadriceps-medial gastrocnemius, *lat QG* lateral quadriceps-lateral gastrocnemius, *NMT* neuromuscular training, *SP* strengtheneing program, *KL* Kellgren Lawrence, *CCI* co-contraction index

### Muscle activation patterns (CCI)

**Fig. 3:**
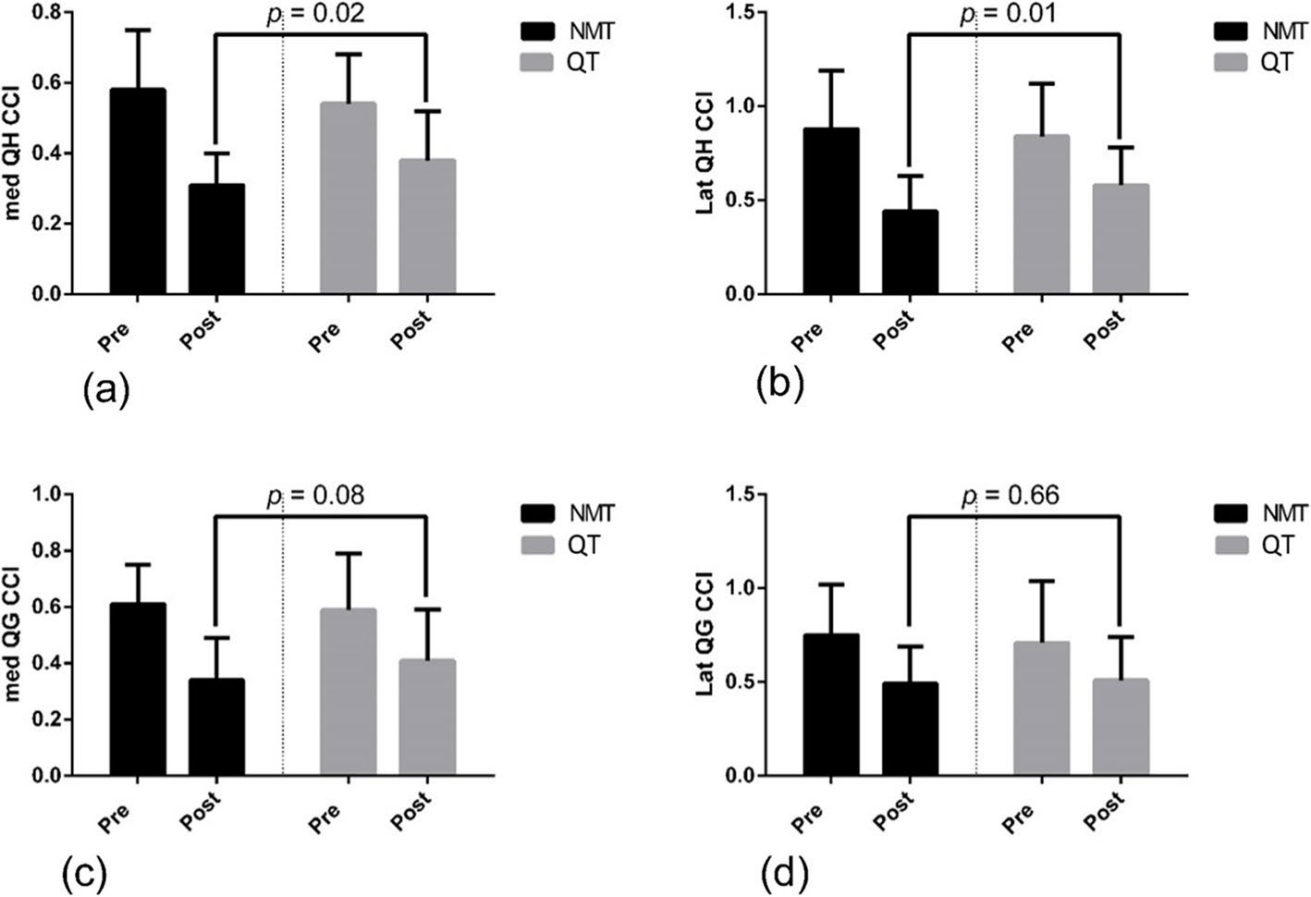
**a-d** Changes in co-contraction index before and after NMT and SP in knee osteoarthritis patients. NMT: neuromuscular training; ST: strengthening program; med QH: medial quadriceps-hamstring; Lat QH: lateral quadriceps-hamstring; med QG: medial quadriceps-gastrocnemius; Lat QG: lateral quadriceps-gastrocnemius

A significant difference was observed between NMT group versus QT group (*p* = .02) for med QHCCI post-intervention period. Similarly for CCI of lat QH (Lat QH), NMT was found to be significantly more effective than QT (*p* = .01) (Fig. 3). However, insignificant differences were observed for CCI of med QG (*p* = .08) and lat QG (*p* = .66) between NMT group and QT group post-training (Fig. 3). Within group analysis revealed significant decrease in the CCI of med QH, lat QH, med QG and lat QG in both NMT and QT groups (Table 4).

**Table 4:** Changes in co-contraction index before and after NMT and QT in knee osteoarthritis patients

CCI		NMT (***n*** = 31)	***p***-value	QT (***n*** = 28)	***p***-value	*Effect size (CI)	t (95% CI)
Med QH	Pre	.58 ± .173	<.001	.54 ± .147	<.001		
	Post	0.31 ± .097		.38 ± .146		−.56 (−1.08, −0.04)	(−2.297, 2.297)
Lat QH	Pre	.88 ± .318	<.001	.84 ± .283	<.001		
	Post	.44 ± .196		.58 ± .209		−.68 (−1.21, −0.16)	(− 2.482, 2.482)
Med QG	Pre	.61 ± .148	<.001	.59 ± .202	<.001		
	Post	.34 ± .15		.41 ± .181		−.38 (−0.90, 0.13)	(−1.739, 1.739)
Lat QG	Pre	.75 ± .273	<.001	.71 ± .338	<.001		
	Post	.49 ± .207		.51 ± .237		−.10 (−0.61, 0.41)	(−0.429, 0.429)

*CCI* co-contraction index, *Med QH* medial quadriceps-hamstring, *Lat QH* lateral quadriceps-hamstring, *Med QG* medial quadriceps-gastrocnemius, *Lat QG* lateral quadriceps-gastrocnemius, *NMT* neuromuscular training, *SP* strengthening program, *CI* confidence intervals

*effect size (CI) is for post-intervention values of CCI between the groups

## Discussion

The purpose of the present study was to investigate and compare the effects of 12 weeks NMT program versus QT on CCI of knee musculature. Findings suggested that NMT program was superior to QT in reducing the CCI of med QH and lat QH muscles. However, NMT and QT were found to be equally effective in reducing the CCI of med QG and lat QG muscles.

NMT showed a significantly greater reduction in the CCI of med QH and lat QH than QT which suggests that it may result in a reduction in articular loads on the knee joint (Fig. 3). A significant reduction in knee joint pain and physical function was also observed in these subjects [24] which further proves the efficacy of NMT in knee OA patients. These results are in accordance with a previous investigation [35] where neuromuscular re-education program was found to be effective in reducing co-contraction of knee muscles in patients with knee OA. Enhanced CCI after NMT observed in the previous [35] and the present study could be attributed to improved sensory motor control i.e. ability to produce controlled movement through coordinated muscle activity and compensatory functional stability in response to NMT^35.^ It could be speculated that these neuromuscular adaptations in response to NMT reduces the stability demands on the knee musculature and thus reduced co-contraction even more than conventional strengthening exercises in the present study. There was a significant reduction in the co-contraction index of med QG and lat QG in both NMT and QT groups when compared with their respective baseline values, but NMT was not found to be superior to QT in reducing it significantly which suggests specificity of NMT towards co-contraction of QH (Fig. 3). Our data showed that NMT led to a significantly greater reduction in co-contraction of medial and lateral knee muscles than QT which indicates that NMT may reduce articular loads on the knee joint and this may have a long term protective effect, reducing the rate of joint destruction. Previous research has also demonstrated similar findings with reduced co-contraction of knee musculature after NMT program in subjects with ACL injury [36]. Interestingly, dynamic NMT has also been shown to improve knee muscle activity just prior to landing in young healthy women [22]. Moreover, in accordance with the findings of present study, Preece et al. [35] also demonstrated reduced medial co-contractions during the pre-contact phase of gait in response to 20 sessions of neuromuscular exercises in knee OA patients. Furthermore, as short as 6 weeks NMT program has also shown significant decline in CCI of knee musculature in knee OA patients demonstrating some sort of neuromuscular remodeling in response to NMT [37]. In the present investigation, CCI of med and lat QG was reduced by both NMT and QT. NMT did not led to any additional improvements in the QG CCI which indicates the specificity of NMT towards quadriceps and hamstring group of muscles. Knee OA patients exhibit sensory-motor deficiencies at different levels of the sensory motor system, from sensory input through integration and processing of information in the central nervous system (CNS) to motor output to perform voluntary movements and maintain postural control. It is assumed that this sensori-motor dysfunction could contribute to development and progression of degeneration in the knee [38, 39]. NMT seems to improve sensory motor control and achieve compensatory functional stability which reduces the need for additional dynamic muscular stability or co-contractions [40]. Moreover, the reduction in CCI of knee muscles could also be attributed to a significant reduction in pain and other symptoms of knee OA in response to NMT as demonstrated in the primary analysis of this study [24].

Within group analysis revealed that QT was also effective in reducing the CCI of opposing muscles of the knee joint which suggests that strength of quadriceps muscle seems to be an important factor in reducing undesirable co-contractions in knee OA with varus malalignment [41, 42]. However, it is to be noticed that using it solely is not justifiable as it is not only the strength of quadriceps muscle that contributes to the degeneration in the joint and rather other factors such as sensory motor deficits and dynamic stability of the knee joint which plays a crucial role in the progression of OA [43].

The present study suffered from certain limitations. The present study was not adequately powered to identify the course of disease progression. Therefore, we recommend that long-term effects of the NMT and QT should be identified by future research. Moreover, small sample size is another limitation of this study which compels us to recommend this study on a larger sample for more conclusive findings. There was a lack of non-exercise control group for proper understanding of the progression of the disease in the present study which could have enabled us to track the potential effect of time on outcome measures. Although every possible measure was taken to obtain clear signal, cross talks during treadmill walking might have contaminated the EMG signal. Since the present sample constituted mild to moderate knee OA, findings of this study could not be applicable to patients with severe knee OA. Further research should be conducted in order to elucidate the effect of different NMT exercise protocols on relevant clinical outcomes in OA knee so as to design an optimal NMT program for these patients. Despite these limitations, it is one of a few clinical trials on the effects of NMT on muscle activation patterns in knee OA patients with varus malalignment conducted with rigorous methodology and gives immense contribution to the existing literature on the physiotherapeutic management of knee OA.

## Conclusion

Findings of this study demonstrated that NMT led to a significantly greater reduction in CCI of knee muscles (med QH and lat QH) than the conventional QT. These findings suggest that NMT could be considered in the rehabilitation of knee OA for better neuromuscular control and reduced knee joint loading.

## Data Availability

Can be provided on request basis.

## Abbreviations

NMT: Neuromuscular Training
QT: Quadriceps Training
CCI: Co-contraction Index
OA: Knee Osteoarthritis
EMG: Electromyography
Med QH: Medial Quadriceps-Medial Hamstring
Lat QH: Lateral Quadriceps-Lateral Hamstring
Med QG: Medial Quadriceps-Medial Gastrocnemius
Lat QG: Lateral Quadriceps and Lateral Gastrocnemius
MVC: Maximum Voluntary Muscle Contractions.
KL Grade: Kellgren-Lawrence Grade
AgCl: Silver-Chloride
1RM: One Repetition Maximum
CNS: Central Nervous System
